# IoT-RiceMobileNet: An improved lightweight MobileNetV2 Model for real-time multi-class rice disease detection using IoT

**DOI:** 10.1371/journal.pone.0356383

**Published:** 2026-09-15

**Authors:** Khawja Imran Masud, Md Yasin Zihad, Mehedi Hasan Shuvo, Mst Raonik Jannat, Jia Uddin, Sahara Ali

**Affiliations:** 1 Department of Computer Science and Engineering, Dhaka University of Engineering & Technology, Gazipur, Bangladesh; 2 AI and Big Data Department, Endicott College, Woosong University, Daejeon, South Korea; 3 Anuradha and Vikas Sinha Department of Data Science, University of North Texas, Denton, Texas, United States of America; Lincoln University College, MALAYSIA

## Abstract

Early and real-time detection of rice leaf diseases (RLD) poses a significant challenge for farmers, especially in rural regions with limited access to advanced technology. Conventional deep learning models often require substantial computational resources, rendering them impractical for deployment on mobile or edge devices commonly used in agricultural environments. Although models such as ResNet50, VGG16, and InceptionV3 can achieve high accuracy, they are computationally expensive and may be less suitable for real-time deployment in smart agricultural systems. Furthermore, many existing studies rely on limited datasets, which can restrict model generalizability under diverse real-world conditions. Although transfer learning can improve classification performance, developing lightweight models suitable for real-time IoT deployment remains challenging. To address these limitations, we curated a hybrid dataset of 7,092 rice leaf images by combining self-collected and Kaggle samples and proposed IoT-RiceMobileNet, a lightweight improved MobileNetV2-based model that can be effectively integrated into our developed IoT system. The proposed model outperformed transfer learning and deep learning baseline models, achieving 99.19% test accuracy and 99.20% precision while maintaining low computational complexity. We further confirmed model stability using stratified 5-fold and 10-fold cross-validation on both the constructed RLD and multi-source datasets, achieving mean accuracies of 98.04% and 98.13% on the constructed RLD dataset and 98.11% and 98.58% on the multi-source dataset, respectively. Moreover, our models compact size and 85.17 FPS inference speed support real-time deployment. Finally, the model was integrated into an IoT-enabled mobile, web, and cloud-based inference framework, demonstrating its practical potential for scalable rice disease detection in smart agriculture.

## Introduction

Rice is a major staple crop that supports food security for a large proportion of the global population, particularly in Asia and Africa [1]. It is deeply embedded in the cultures, diets, and economies of many developing countries, including Bangladesh, and provides an important source of carbohydrates, vitamins, and minerals [2,3]. Rice plants are affected by several diseases, including bacterial leaf blight, brown spot, leaf blast, leaf scald, and narrow brown spot, which can reduce growth and yield and spread across plantations, causing substantial economic losses for farmers [4–6]. Recent advances in machine learning and deep learning have created new opportunities for automated, scalable, and accurate rice leaf disease detection under diverse crop-growing conditions [7–11]. Computer vision-based approaches, particularly convolutional neural networks (CNNs), have shown strong capability in extracting discriminative visual features from leaf images for fast and accurate disease classification [12–15]. Transfer learning (TL) has further accelerated progress in this area by adapting large pre-trained models to domain-specific disease classification tasks, reducing training time and computational cost [16,17]. In recent years, Internet of Things (IoT) [18] based smart agricultural systems have evolved from simple offline image classifiers to connected, real-time decision-support platforms that integrate cameras, sensors, edge devices, and cloud servers to deliver timely disease predictions to farmers [19–21]. The convergence of deep learning and IoT has been widely recognized as a promising approach for image-based crop disease monitoring, remote diagnosis, and automated classification [22,23]. However, practical deployment under real field conditions requires models that are not only accurate but also lightweight and computationally efficient enough for resource-constrained agricultural environments [24].

Scholarly inquiries into rice leaf disease detection using ML- and DL-based models have progressively evolved. On the machine learning side, Sethy et al. [25] combined ResNet50-based deep features with SVM and achieved promising performance; however, their study was limited to four disease classes and did not include real-world deployment. Chaudhary et al. [26] used GLCM and ILMFD-based feature representations with SVM and ANN, reporting competitive results, but the approach remained dependent on hand-crafted features and a limited disease scope. Narmadha et al. [27] reported 97.68% accuracy using only 120 images from three disease classes, which raises concerns regarding generalizability. Li et al. [28] proposed a RegNet-based model for detecting 11 rice disease classes and one healthy class, achieving 96.8% accuracy. Li et al. [29] trained an RDRM-YOLO model on approximately 5,930 images from four disease classes and reported 94.3% accuracy, while Al et al. [30] reported 98.71% accuracy using 2,368 images from four disease classes. Although these studies achieved promising classification performance, many were limited by fewer disease classes, smaller datasets, limited deployment analysis, or insufficient evaluation under diverse real-world conditions. Haruna et al. [31] investigated a StyleGAN2-ADA-enhanced rice leaf disease detection framework using Faster R-CNN and SSD, achieving up to 93% mAP under small-data conditions. Simhadri et al. [32] evaluated 15 pre-trained CNN architectures and reported 99.64% accuracy with InceptionV3; however, the study was conducted on controlled datasets and relied on computationally expensive architectures, limiting suitability for edge deployment. Poorni et al. [33] applied transfer learning using a pre-trained InceptionV3 model and achieved 94.48% accuracy, but the study had limited real-world validation. For IoT-oriented deployment, Sriram et al. [34] integrated InceptionV3 with an IoT-based irrigation control system on Raspberry Pi and achieved 98.12% accuracy using real-field rice images. Verma et al. [35] developed a smartphone- and FPGA-integrated CNN framework for rice and potato disease quantification, achieving 95% classification accuracy. However, these systems were still constrained by limited dataset scale, restricted disease coverage, or incomplete evaluation of lightweight real-time deployment requirements.

Overall, existing studies have advanced rice disease classification using machine learning, deep learning, transfer learning, and IoT-based systems. However, several limitations remain. Many studies rely on relatively small or less diverse datasets, evaluate only a limited number of disease classes, or use computationally expensive architectures that are difficult to deploy on resource-constrained mobile and IoT devices. In addition, relatively limited work has jointly considered dataset diversity, lightweight model design, cross-source validation, explainability, and end-to-end IoT deployment within a unified rice leaf disease detection framework. To address these limitations, this study introduces IoT-RiceMobileNet with the following key contributions:

1. A self-curated constructed RLD dataset of 7,092 rice leaf images across six classes, including five disease classes and healthy leaves, designed to improve real-world generalization.
2. A lightweight IoT-RiceMobileNet model built on MobileNetV2, achieving 99.19% test accuracy and 99.20% precision on the constructed RLD dataset while maintaining low computational requirements.
3. A comprehensive evaluation against eight widely used deep learning architectures on the constructed RLD dataset, supported by stratified cross-validation and additional evaluation on a heterogeneous multi-source dataset of 14,758 images, where IoT-RiceMobileNet achieved 99.25% test accuracy and demonstrated strong cross-source generalization.
4. An end-to-end IoT system for real-time rice leaf disease classification, integrating the proposed model with mobile, web, cloud, and hardware components.

## Materials and methods

A complete IoT-based solution was designed and developed for real-time rice leaf disease detection using the proposed lightweight MobileNetV2-based IoT-RiceMobileNet architecture.

**Fig 1:**
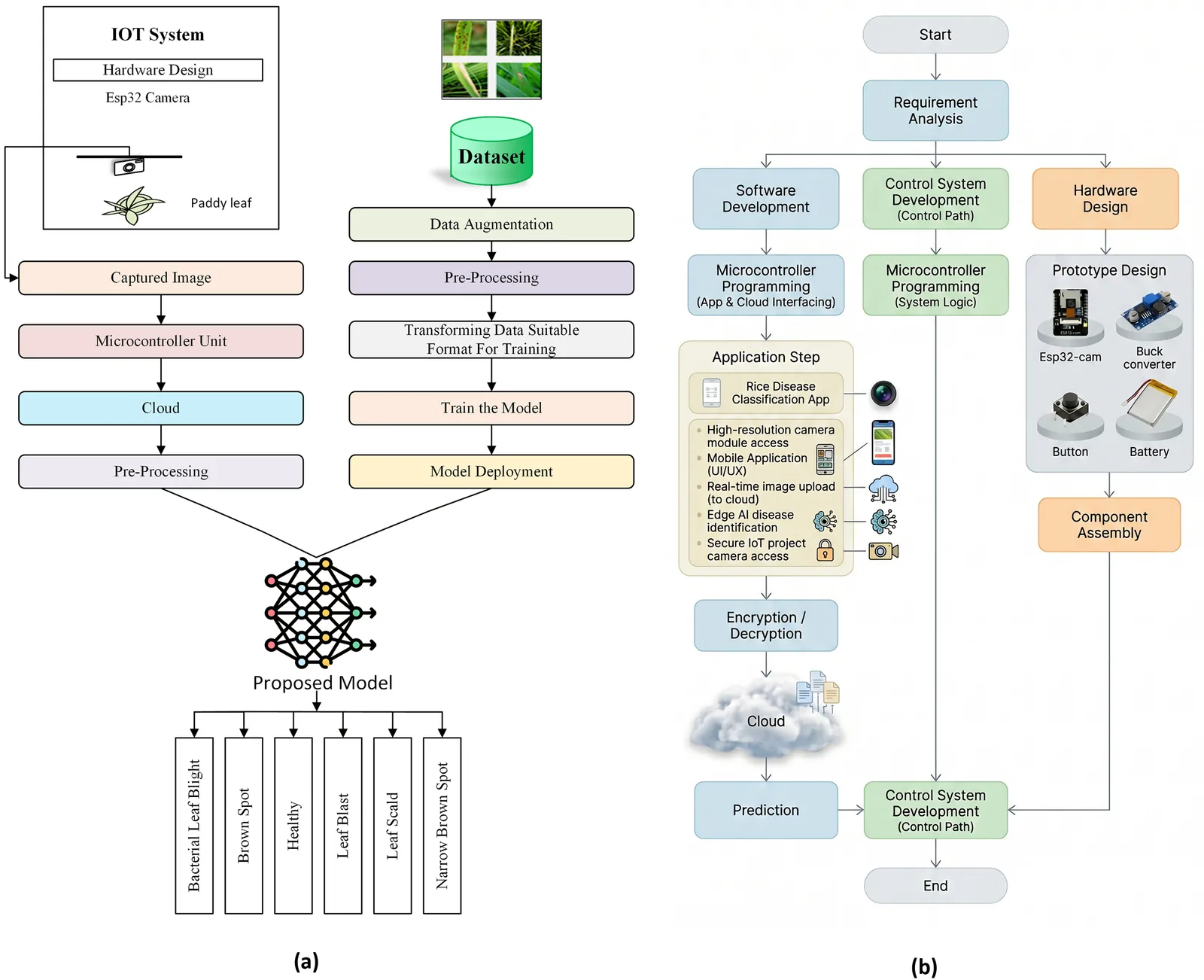
Overall architectural overview of the proposed system. (a): IoT-RiceMobileNet framework. (b): IoT system development workflow.

As illustrated in Fig 1, the framework consists of two connected workflows: model development and IoT-based inference. In the model development workflow, rice leaf images are collected from the dataset, augmented, preprocessed, transformed into a suitable training format, and used to train and deploy the proposed model. In the IoT-based inference workflow, leaf images captured through the ESP32-CAM hardware module are processed by the microcontroller and transmitted to the cloud, where the same preprocessing procedure is applied before classification. The deployed model then predicts one of the six rice leaf classes and delivers the result through the mobile or web application.

### Dataset description

**Fig 2:**
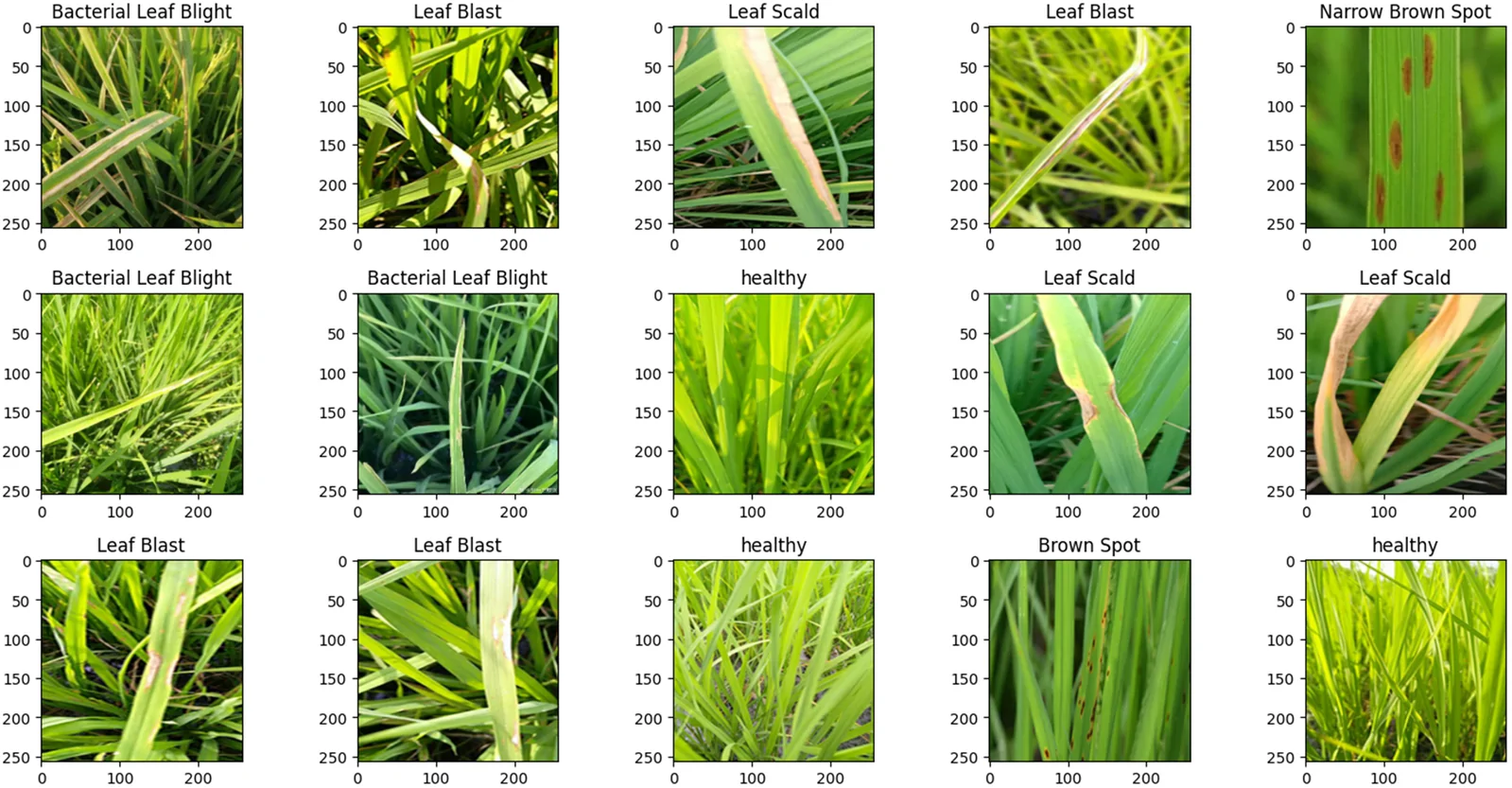
Representative samples of the six rice leaf classes used in the dataset. The classes include bacterial leaf blight, leaf blast, leaf scald, narrow brown spot, brown spot, and healthy leaves.

This study developed a rice leaf disease (RLD) dataset comprising six rice leaf conditions, including five disease classes (bacterial leaf blight, brown spot, leaf blast, leaf scald, and narrow brown spot) and healthy leaves. The dataset was constructed by integrating self-collected field images with publicly available Kaggle images [36] to improve environmental diversity and model generalization capability. The original dataset consisted of 3,401 images, including 773 self-collected field images and 2,628 Kaggle images. After augmentation of the self-collected training data and integration with the Kaggle dataset, the final constructed RLD dataset contained 7,092 images across six classes. The overall dataset construction, augmentation statistics, and partitioning strategy are summarized in Tables 1 and 2, while representative samples of all six classes are illustrated in Fig 2.

**Table 1:** Self-collected field dataset split and augmentation statistics.

Class	Collected Samples	Self Train Originals	Self Val./Test Originals	Self Train After Aug.
bacterial_leaf_blight	116	58	58	687
brown_spot	190	95	95	912
healthy	110	55	55	720
leaf_blast	133	66	67	763
leaf_scald	107	53	54	605
narrow_brown_spot	117	59	58	390
Total	773	386	387	4077

**Table 2:** Final composition of the constructed RLD dataset.

Class	Kaggle Total [36]	Kaggle Train	Self Train After Aug.	Train Total	Val. Total	Test Total	Train + Val. + Test
bacterial_leaf_blight	438	226	687	913	135	135	1183
brown_spot	438	236	912	1148	148	149	1445
healthy	438	252	720	972	120	121	1213
leaf_blast	438	253	763	1016	126	126	1268
leaf_scald	438	270	605	875	111	111	1097
narrow_brown_spot	438	289	390	679	104	103	886
Total	2628	1526	4077	5603	744	745	7092

#### Self-collected image acquisition.

A total of 827 rice leaf images were collected from approximately 14 agricultural fields in Chapulia, Gazipur, Bangladesh, during the Aman 2025 growing season using an iPhone 12 Pro under natural daylight conditions. No formal collection permit was required because the images were collected from privately accessible agricultural fields with permission from the field owners. No destructive sampling, endangered species, protected plant materials, human participants, or animals were involved in this study. Images were captured at different times of day (morning, midday, and afternoon) to incorporate variations in illumination and shadow effects. Multiple viewpoints and distances were used to reflect real-world farmer-acquired field conditions. Prior to annotation, all images underwent manual quality screening to remove samples affected by severe blur, overexposure, occlusion, or insufficient disease visibility. After screening, 54 images were excluded, resulting in 773 high-quality images for annotation and analysis. Images were first separated into healthy and diseased categories, after which diseased samples were assigned to specific disease classes based on dominant visible symptoms. Disease classification followed standardized visual diagnostic criteria for bacterial leaf blight, brown spot, leaf blast, leaf scald, narrow brown spot, and healthy leaves, guided by the IRRI Rice Knowledge Bank and the Rice Doctor application. All self-collected images were annotated at the class level with the assistance of two agricultural experts in rice disease diagnosis and plant pathology. Ambiguous or overlapping cases were independently reviewed by both experts, and samples without diagnostic consensus were excluded. Final labeling decisions were verified under the supervision of the corresponding author.

#### Image preprocessing, augmentation, and dataset partitioning.

Image preprocessing and augmentation techniques were applied to improve robustness and reduce overfitting [37,38]. All images were resized to 224×224 pixels to meet the input requirements of MobileNet-based architectures and ensure consistent dimensionality across the dataset. Pixel intensity values were normalized from [0,255] to [0,1] to improve optimization stability and accelerate model convergence [39]. To address limited dataset size and class imbalance, a comprehensive augmentation strategy was applied only to the training portion of the self-collected dataset. This included geometric transformations: random rotation, horizontal and vertical flipping, translation, zooming, and shearing and photometric transformations such as brightness and contrast adjustments [37,38,40,41]. This process increased intra-class diversity and improved model robustness to real-world field conditions while reducing overfitting to specific acquisition settings and preserving disease-specific visual characteristics. As shown in Table 1, 386 original self-collected images were used for training augmentation, producing 4,077 self-collected training samples after augmentation, while 387 original self-collected images were reserved for validation and testing.

**Fig 3:**
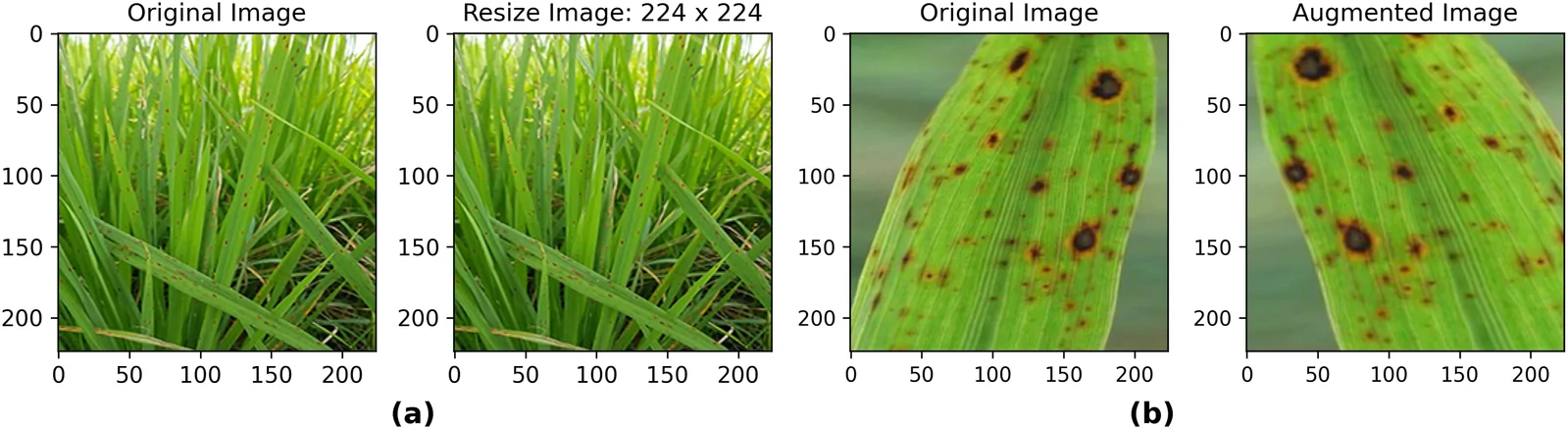
Comparison of image preprocessing and augmentation steps. (a): Original and resized images. (b): Original and augmented images.

The augmentation and preprocessing pipeline is illustrated in Fig 3. Original self-collected images were partitioned at the source-image level into training and evaluation subsets before any augmentation, ensuring that each source image and its augmented derivatives belonged exclusively to a single partition. Augmentation was applied only to the self-collected training subset. The augmented self-collected dataset was merged with the publicly available Kaggle rice leaf disease dataset [36]. From the Kaggle dataset, 1,526 images were used for training and 1,102 images were reserved for the validation/test holdout set. The final training partition contained 5,603 images, consisting of 1,526 Kaggle training images and 4,077 augmented self-collected training images. The holdout partition contained 1,489 original images, consisting of 1,102 Kaggle images and 387 self-collected images. The holdout partition was then divided into validation and test subsets, resulting in 744 validation images and 745 test images, as reported in Table 2. The validation subset was used for hyperparameter tuning and model selection, whereas the test subset was reserved exclusively for final performance evaluation. Because validation and test images were kept unaugmented, the final performance evaluation was conducted on unseen original images rather than augmented derivatives.

#### Additional multi-source benchmark dataset.

In addition to the constructed RLD dataset, a heterogeneous multi-source benchmark dataset was prepared using publicly available Kaggle [42] and Mendeley [43] rice leaf disease images.

**Fig 4:**
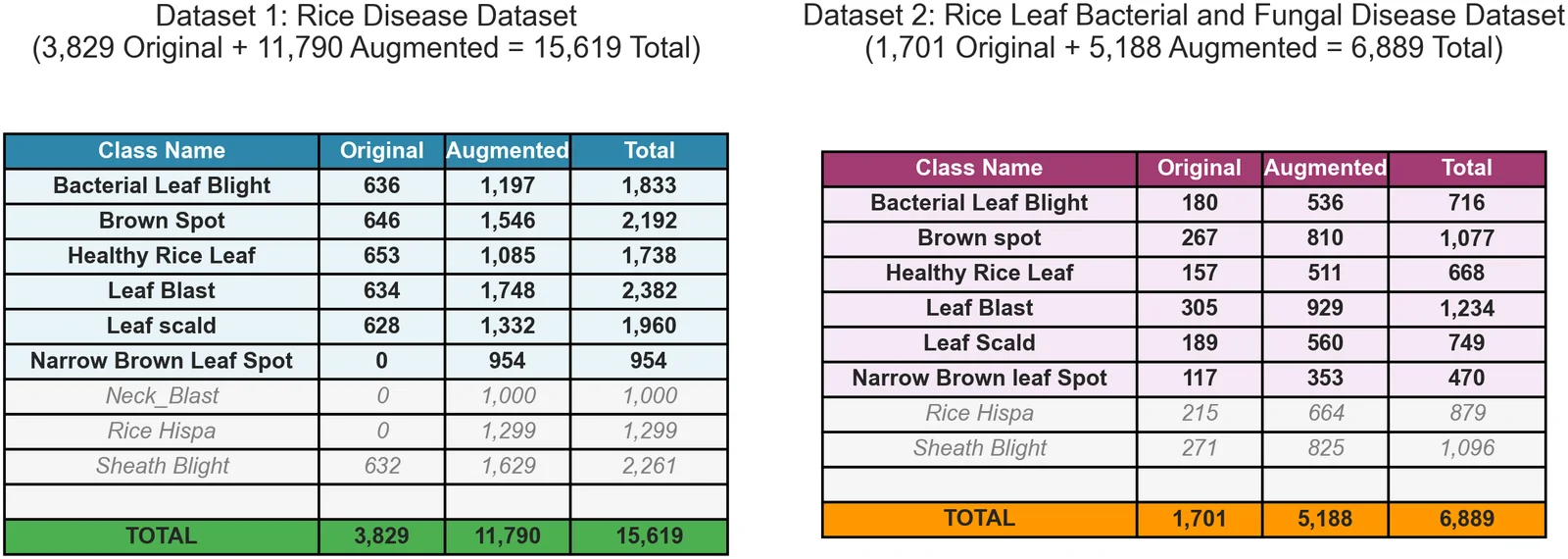
Multi-source dataset integration and partitioning details. The figure summarizes the integration of Kaggle and Mendeley rice leaf disease images and their partitioning into training, validation, and testing subsets.

This dataset was used for additional training, validation, and testing experiments to evaluate model robustness across different acquisition conditions, backgrounds, disease appearances, and dataset preparation pipelines. The multi-source dataset statistics are shown in Fig 4.

### Model development

We proposed a lightweight improved MobileNetV2 architecture for real-time rice leaf disease classification and systematically evaluated its performance against several deep learning baselines. We conducted comparative experiments across eight baseline models, encompassing canonical CNN, VGG16, MobileNetV2, InceptionV3, and ResNet50, with selected architectures augmented through transfer learning paradigms to assess the impact of pre-trained weights.

#### Convolutional neural network (CNN).

**Fig 5:**
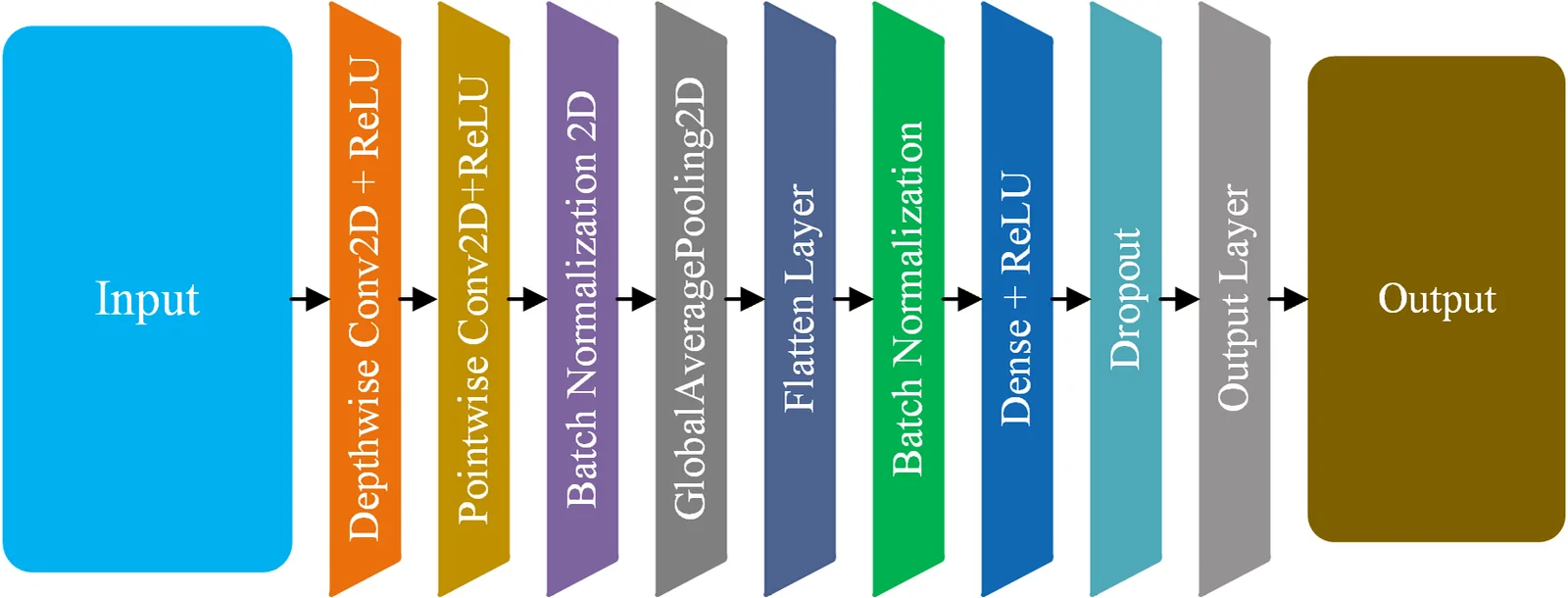
Architecture of the CNN baseline model.

For fair comparison with our models, we developed a baseline convolutional neural network (CNN) model and trained it using the same dataset partitions. CNNs are effective at learning complex spatial patterns from image data and have significantly advanced image classification and object detection tasks [44]. Operating through multiple layers, convolutional, pooling, and fully connected layers, CNNs systematically extract and build hierarchical feature representations from raw images. Their strength stems from local feature detection and compositional learning, which culminate in superior performance for object detection and image classification tasks [32],. The CNN architecture used in our experiment is shown in Fig 5.

#### Transfer learning.

Transfer learning uses features learned from one task to improve performance on a related but distinct task, accelerating convergence and improving classification accuracy through feature reuse [45].

**Fig 6:**
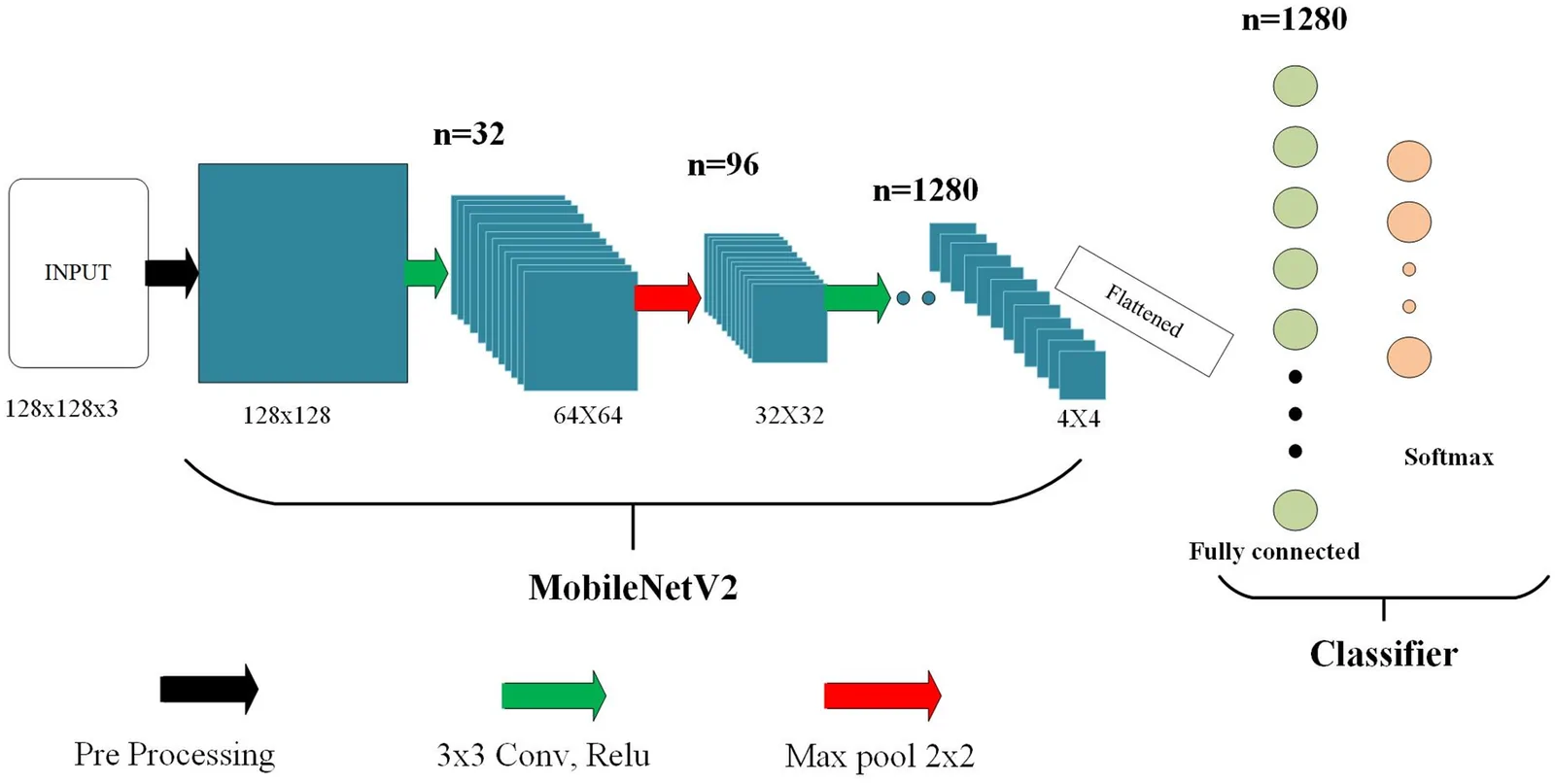
Transfer learning architecture using MobileNetV2. The figure illustrates the MobileNetV2-based transfer learning workflow used for rice leaf disease classification.

Two important paradigms exist: feature extraction, wherein only terminal layers undergo retraining while the pre-trained model’s weights remain frozen, and fine-tuning, which refurbishes select higher-level layers of the architecture on novel data [45]. Pre-trained models can reduce training time and improve generalization by extracting meaningful feature representations from new samples, which is particularly salient for tasks plagued by scarce labeled data, and researchers frequently use this technique [46]. By learning models pre-trained on voluminous datasets such as ImageNet [47], practitioners can judiciously calibrate them for domain-specific applications [48]. Therefore, to develop our model, we employed a pre-trained MobileNetV2 architecture and then customized the layer architecture and systematically experimented with diverse transfer learning methodologies, achieving strong classification of rice leaf diseases through feature extraction and fine-tuning strategies. The MobileNetV2-based transfer learning workflow is shown in Fig 6.

#### Proposed IoT-RiceMobileNet model.

**Fig 7:**
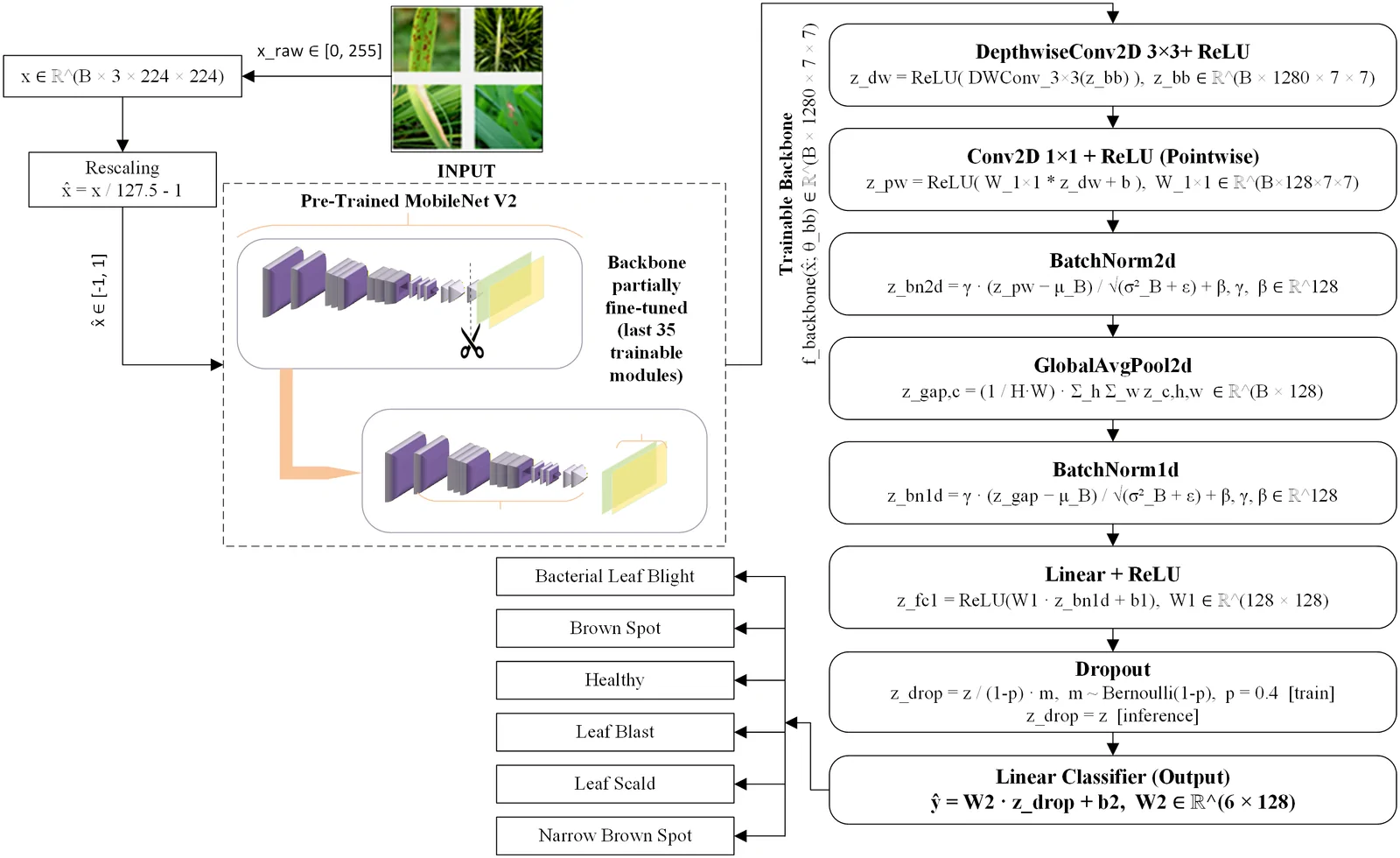
Proposed IoT-RiceMobileNet model architecture. The architecture illustrates the MobileNetV2 backbone, customized lightweight convolutional layers, and final classification module for six rice leaf classes.

In this study, we developed IoT-RiceMobileNet, a MobileNetV2-based lightweight CNN architecture designed for IoT-based real-time multi-class rice disease detection. The proposed architecture, shown in Fig 7, uses a MobileNetV2 backbone pre-trained on the ImageNet dataset to accelerate convergence and improve performance on the domain-specific constructed RLD dataset. The top classification layers of MobileNetV2 were discarded, and the image input shape was set to (224, 224, 3). To adapt the pre-trained backbone to rice leaf disease classification, we applied a fine-tuning strategy. Initially, all layers of MobileNetV2 were set as trainable. We then employed a freezing method, where all layers except the last 35 were frozen and tuned as necessary to prevent updating the early layers, which typically learn simple, low-level features such as edges and textures. Pixel values were normalized from the standard range [0, 255] to [−1,1] using:


$$\begin{array}{c} x^{\prime }=2x_{c}-1,\;x_{c}\in [0,1] \end{array}$$ (1)

where $x_{c}=x/255$ is the pixel intensity scaled to [0,1] via standard image-to-tensor conversion in PyTorch, and $x^{\prime }\in [-1,1]$ represents the normalized value used as input to the network. This normalization improves convergence speed and numerical stability in gradient-based optimization. The same normalization procedure is applied consistently during both training and inference. After the input and rescaling layers, the pre-trained MobileNetV2 backbone was incorporated. Custom convolutional layers were added beyond the pre-trained layers to improve feature extraction. The ReLU activation function was used in all hidden layers:


$$\begin{array}{c} \text{ReLU}(z)=\max(0,z) \end{array}$$ (2)

Following the final convolutional block of the backbone network, a depthwise convolution layer (1280→1280, kernel size (3×3)) was employed to further refine spatial feature representations. This was followed by a pointwise convolution layer (1280→128, kernel size (1×1)) to reduce channel dimensionality and computational complexity. Batch normalization (BN2d) was subsequently applied to stabilize feature distributions and facilitate training convergence. A Global Average Pooling (GAP) layer compresses the spatial feature maps into a 128-dimensional feature vector, significantly reducing the number of trainable parameters while improving generalization capability. A second batch normalization layer (BN1d) was then applied to normalize the resulting feature embeddings before classification. The pooled feature vector was passed through a fully connected layer with 128 neurons and ReLU activation to learn non-linear discriminative representations. To mitigate overfitting, a dropout layer with a dropout probability of *p* = 0.4 was employed. Finally, a linear classification layer maps the 128-dimensional feature representation to *K* output class logits, which are converted into class probabilities using the Softmax function:


$$\begin{array}{c} P(y=k|𝐱)=\frac{e^{z_{k}}}{\sum_{j=1}^{K}e^{z_{j}}},\;k=1,2,...,K \end{array}$$ (3)

where $z_{k}$ denotes the logit corresponding to the *k*-th class, and *K* = 6 represents the total number of rice disease categories. In practice, during training, raw logits are optimized using nn.CrossEntropyLoss, while softmax was applied at inference time when probability estimates are required for interpretation.

Overall, IoT-RiceMobileNet combined a pretrained MobileNetV2 backbone with depthwise-separable convolutions, dual batch normalization, global average pooling, and selective fine-tuning of the last 35 layers, forming a compact and efficient architecture for multi-class rice disease classification.

### IoT system development

**Fig 8:**
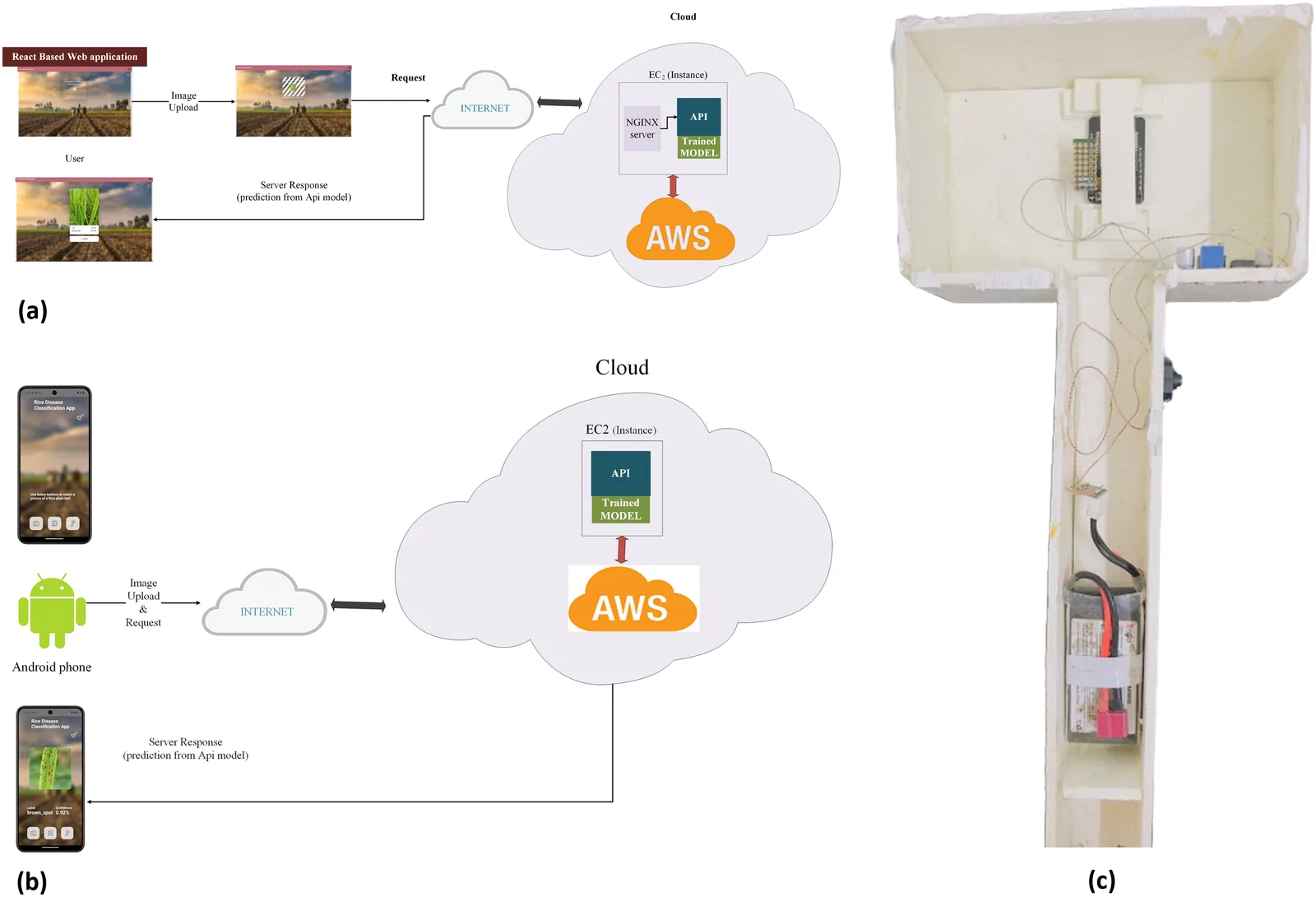
System deployment architecture of the IoT-RiceMobileNet framework. (a): Web-based application flow. (b): Mobile application flow. (c): Hardware system design.

The IoT-based rice disease classification system comprises several integrated stages. Requirements analysis identified system needs and constraints. The software component controls image capture, disease detection, and remote camera access through microcontroller programming. Hardware components include ESP32-CAM, buck converter, button, and rechargeable battery assembled into a compact prototype mounted on a PVC base (Fig 8(c)). The microcontroller transmits captured image data to the cloud for disease prediction and classification. A mobile app was developed for Android using React Native to facilitate accessibility and usability. Users capture leaf images via camera or upload from the gallery, and the app communicates with a backend API on AWS EC2. The API processes images using the trained deep learning model and responds with predictions in real-time. The lightweight pipeline enables low-latency user interaction, facilitating field deployment for farmers and agricultural extension workers. Fig 8(a) shows the web view architecture, and Fig 8(b) shows the mobile app.

#### System deployment and inference pipeline.

The proposed rice disease detection system was deployed as a cloud-based inference service integrated with IoT devices, a mobile application, and a web interface. These client platforms serve as image input sources and communicate with a centralized backend hosted on an AWS EC2 instance.

The backend was implemented using FastAPI and exposes RESTful API endpoints for receiving image requests and returning prediction results. Upon receiving an image, the server validates the input and applies the same preprocessing operations used during model training, including RGB conversion, resizing to 224×224 pixels, tensor conversion, and normalization. The preprocessed image is then forwarded to the pretrained IoT-RiceMobileNet model loaded from a .pth checkpoint file. During inference, the model is set to evaluation mode and executed within torch.no_grad() to disable gradient computation and reduce computational overhead. The model produces raw logits, which are converted into class probabilities using the Softmax function. The class with the highest probability is selected as the final prediction, and the corresponding confidence score is returned to the client in JSON format. This deployment architecture shown in Fig 8 enables real-time, scalable, and device-independent rice disease detection across IoT devices, mobile applications, and web platforms. Algorithm 1 summarizes the complete inference pipeline.

**Algorithm 1: Cloud-based IoT-integrated rice disease detection and classification pipeline**

**Input:** Image *I* submitted from an IoT device, mobile application, or web client

**Output:** Predicted disease class *C* and confidence score *p*

1:  Initialize the FastAPI backend server on the AWS EC2 instance;

2:  Load the trained IoT-RiceMobileNet model *M* from the .pth checkpoint;

3:  Set the model to evaluation mode: M←M.eval();

4:  Receive image *I* through the REST API endpoint;

5:  Validate the image format and request payload;

6:  Convert *I* to RGB format;

7:  Resize *I* to 224×224 pixels;

8:  Normalize *I* using the same preprocessing settings used during training;

9:  Convert the preprocessed image into tensor *T;*

10:  Add batch dimension to obtain input tensor $T_{b}$;

11:  Disable gradient computation using torch.no_grad();

12:  Compute raw model output logits: $Z\leftarrow M(T_{b})$;

13:  Convert logits into class probabilities: P←Softmax(Z);

14:  Determine the predicted class index: k←argmax(P);

15:  Map class index *k* to disease class label *C;*

16:  Extract confidence score: p←max(P);

17:  Return JSON response containing {*C*,*p*} to the client;

### Evaluation metrics and validation methodology

To evaluate the effectiveness of the proposed IoT-RiceMobileNet model and compare it with baseline architectures, we used standard classification metrics commonly employed in deep learning-based image classification. These metrics provide a comprehensive assessment of the model’s ability to classify rice leaf diseases accurately. We employed a multi-source dataset methodology to demonstrate the model’s resilience and generalizability.

### Evaluation metrics

The following evaluation metrics were used to assess model performance:

1. Accuracy: Measures the proportion of correct predictions across all test samples:

$$\begin{array}{c} \text{Accuracy}=\frac{TP+TN}{TP+TN+FP+FN} \end{array}$$ (4)

2. Precision: Quantifies the reliability of positive predictions, indicating how often the model is correct when it predicts a disease class:

$$\begin{array}{c} \text{Precision}=\frac{TP}{TP+FP} \end{array}$$ (5)

3. Recall: Measures how well the model identifies actual positive cases. In disease detection, high recall is critical because missing a disease could have serious agricultural consequences:

$$\begin{array}{c} \text{Recall}=\frac{TP}{TP+FN} \end{array}$$ (6)

4. F1-Score: Balances precision and recall by computing their harmonic mean.

$$\begin{array}{c} \text{F1-Score}=2\times \frac{\text{Precision}\times \text{Recall}}{\text{Precision}+\text{Recall}} \end{array}$$ (7)

In the equations above, TP, TN, FP, and FN represent true positives, true negatives, false positives, and false negatives. We also created confusion matrices to show per-class performance and calculated ROC-AUC curves to measure class separation. Gradient-weighted Class Activation Mapping (Grad-CAM) was used to verify whether the model focused on disease-relevant leaf regions.

### Dual-dataset validation strategy

To evaluate generalization, IoT-RiceMobileNet was tested on two dataset settings: the constructed RLD dataset of 7,092 images and an additional heterogeneous multi-source dataset of 14,758 images. The multi-soruce datasets are integrated from Kaggle [42] and Mendeley [43]. This dual-validation strategy reduces dataset bias and demonstrates that the model performs consistently across different data sources and image capture conditions.

## Experiment results and analysis

### Experimental setup

All experiments were conducted on a Windows-based workstation with high-performance computing capacity, including an Intel Core Ultra 9 285K processor running at 3.70 GHz, 64 GB RAM, and a 32 GB GPU. PyTorch was used for model implementation. To ensure reproducibility, the random seed was set to 42, and deterministic PyTorch settings were used across all experiments. Supplementary visualization and model parameter analysis scripts were executed on a separate Windows 11 Pro machine equipped with an Intel Core i5-12400 CPU and 24 GB RAM. All models were evaluated on a held-out test set and compared against multiple baseline and transfer learning configurations. Table 3 summarizes the architectural configurations and training settings of all evaluated models. All models share a common optimization framework, differing only in backbone architecture, fine-tuning strategy, and classification head design.

**Table 3:** Training and architectural configuration of baseline and proposed models.

Category	Component	Configuration
**Training configuration applied to all models**		
Training	Optimization	Adam optimizer ($lr=1\times 10^{-5}$), ReduceLROnPlateau (factor = 0.5, patience = 3), balanced class weights, cross-entropy loss with label smoothing = 0.1.
Training	Regularization	Early stopping (patience = 7); mixed-precision training (AMP) enabled on CUDA devices.
**Baseline models without transfer learning**		
Without TL	ResNet50	Random initialization; all layers trainable; FC → 6 classes.
Without TL	VGG16	Random initialization; all layers trainable; GAP → FC(512) → ReLU → Dropout(0.5) → FC(6).
Without TL	MobileNetV2	Random initialization; all layers trainable; Dropout(0.3) → FC(6).
Without TL	InceptionV3	Random initialization; all layers trainable; FC → 6 classes; aux_logits = False.
**Transfer learning baselines**		
Transfer Learning	ResNet50	ImageNet pretrained; layer4 unfrozen; FC → 6 classes.
Transfer Learning	VGG16	ImageNet pretrained; features [24:] unfrozen; GAP → FC(512) → ReLU → Dropout(0.5) → FC(6).
Transfer Learning	MobileNetV2	ImageNet pretrained; features [18] unfrozen; Dropout(0.3) → FC(6).
Transfer Learning	InceptionV3	ImageNet pretrained; Mixed_7c unfrozen; FC → 6 classes; aux_logits = False.
**Proposed model**		
IoT-RiceMobileNet	MobileNetV2 with Customized Layers	ImageNet-pretrained MobileNetV2 backbone with last 35 leaf modules unfrozen; Depthwise Conv(1280→1280, 3×3) → Pointwise Conv(1280→128) → BN → GAP → BN → FC(128) → ReLU → Dropout(0.4) → FC(6).

Note: TL = Transfer Learning; FC = fully connected layer; GAP = global average pooling; BN = batch normalization; AMP = automatic mixed precision.

### Comparative analysis of baseline models using RLD dataset

We benchmarked IoT-RiceMobileNet against multiple baseline and transfer learning configurations across the training, validation, and testing phases; the results are reported in Table 4. IoT-RiceMobileNet achieved the strongest validation and test performance, with 98.73% training accuracy, the highest validation accuracy of 97.85%, and the highest test accuracy of 99.19%. It also achieved the highest test precision and recall, with 99.20% precision and 99.19% recall, respectively. ResNet50 TL ranked second in test performance, achieving 98.82% training accuracy, 97.58% validation accuracy, and 96.64% test accuracy. These results indicate that IoT-RiceMobileNet provides stronger test-set generalization than the evaluated baseline and transfer learning models while maintaining a lightweight architecture suitable for IoT deployment.

**Table 4:** Comparative analysis of the proposed model against other deep learning models on the RLD dataset.

Model	Training			Validation			Testing		
	Acc	Prec	Rec	Acc	Prec	Rec	Acc	Prec	Rec
CNN	0.5899	0.5875	0.5899	0.6089	0.6088	0.6089	0.6027	0.5837	0.6027
VGG16	0.9381	0.9381	0.9381	0.9435	0.9440	0.9435	0.9557	0.9560	0.9557
VGG16 TL	0.9614	0.9616	0.9614	0.9462	0.9474	0.9462	0.9490	0.9498	0.9490
MobileNetV2	0.7087	0.7154	0.7087	0.7070	0.7222	0.7070	0.7221	0.7368	0.7221
MobileNetV2 TL	0.8606	0.8638	0.8606	0.8293	0.8343	0.8293	0.8416	0.8495	0.8416
InceptionV3	0.7494	0.7504	0.7494	0.8723	0.8764	0.8723	0.8577	0.8603	0.8577
InceptionV3 TL	0.9154	0.9165	0.9154	0.9207	0.9219	0.9207	0.9101	0.9116	0.9101
ResNet50	0.8188	0.8234	0.8188	0.8118	0.8287	0.8118	0.8268	0.8453	0.8268
ResNet50 TL	0.9882	0.9882	0.9882	0.9758	0.9760	0.9758	0.9664	0.9666	0.9664
IoT-RiceMobileNet	0.9873	0.9873	0.9873	0.9785	0.9790	0.9785	0.9919	0.9920	0.9919

Note: TL = Transfer Learning. For models trained with checkpoint selection, training and validation metrics were reported from the epoch corresponding to the lowest validation loss, while test metrics were computed using the selected best-performing checkpoint.

**Fig 9:**
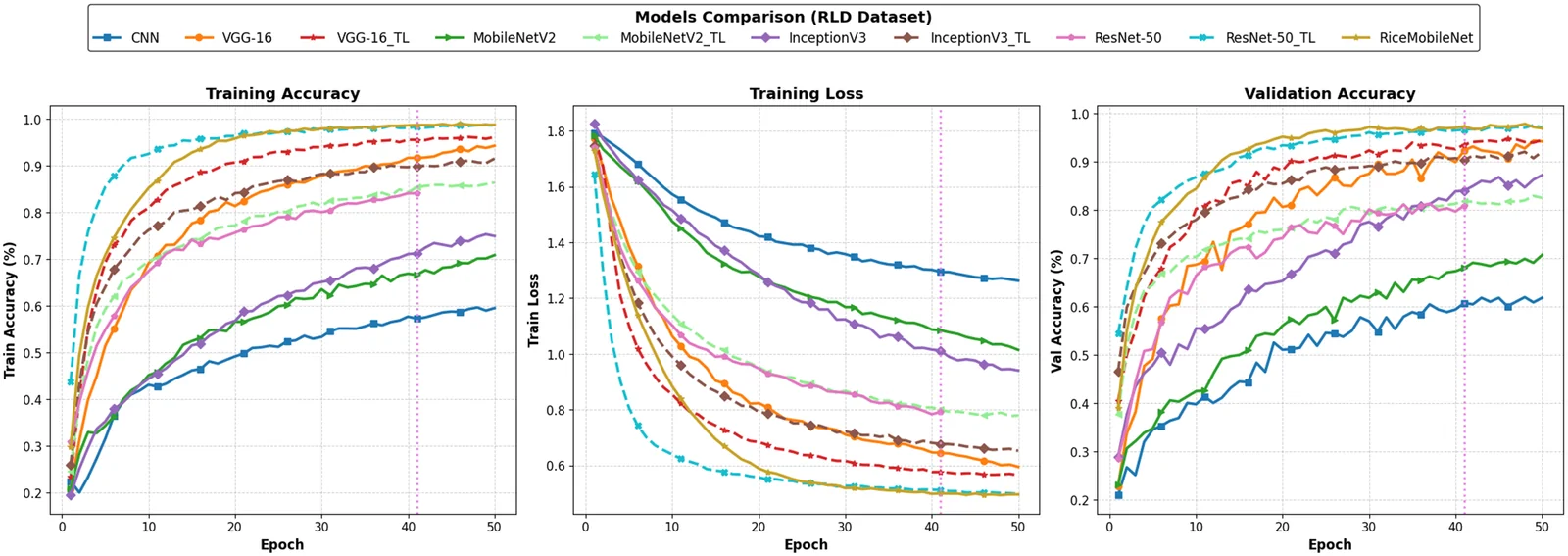
Model-wise training and validation performance across all architectures on the RLD dataset. The figure compares training accuracy, training loss, and validation accuracy over 50 epochs. Models were trained using early stopping and checkpoint selection based on validation loss.

Overall, transfer learning improved performance for most architectures, particularly ResNet50, MobileNetV2, and InceptionV3. ResNet50 TL achieved 96.64% test accuracy, substantially outperforming scratch-trained ResNet50, which achieved 82.68%. InceptionV3 TL and MobileNetV2 TL achieved 91.01% and 84.16% test accuracy, respectively, also outperforming their scratch-trained counterparts. However, scratch-trained VGG16 achieved 95.57% test accuracy, slightly outperforming VGG16 TL. These results indicate that the effectiveness of transfer learning varies across architectures, while IoT-RiceMobileNet achieved the strongest overall test-set performance with 99.19% accuracy. Fig 9 also shows the effectiveness of the proposed models during training.

### Comparative analysis of baseline models using multi-source dataset

To further assess the robustness and generalizability of IoT-RiceMobileNet, additional experiments were conducted on a multi-source dataset comprising 14,758 rice leaf images collected from Kaggle [42] and Mendeley [43]. The dataset spans six rice disease categories and provides an additional benchmark with greater source diversity than the RLD dataset. Comparative results are presented in Table 5.

**Table 5:** Performance comparison of different deep learning models on the multi-source dataset.

Model	Training			Validation			Testing		
	Acc	Prec	Rec	Acc	Prec	Rec	Acc	Prec	Rec
CNN	0.6391	0.6536	0.6391	0.6606	0.6676	0.6606	0.6484	0.6482	0.6484
VGG16	0.9226	0.9258	0.9226	0.9383	0.9419	0.9383	0.9404	0.9423	0.9404
VGG16 TL	0.9481	0.9493	0.9481	0.9282	0.9308	0.9282	0.9214	0.9250	0.9214
MobileNetV2	0.7175	0.7318	0.7175	0.7561	0.7609	0.7561	0.7425	0.7465	0.7425
MobileNetV2 TL	0.7570	0.7693	0.7570	0.7229	0.7346	0.7229	0.7290	0.7372	0.7290
InceptionV3	0.7472	0.7600	0.7472	0.8449	0.8548	0.8449	0.8381	0.8501	0.8381
InceptionV3 TL	0.8635	0.8675	0.8635	0.8862	0.8940	0.8862	0.8814	0.8878	0.8814
ResNet50	0.9177	0.9199	0.9177	0.9275	0.9301	0.9275	0.9329	0.9373	0.9329
ResNet50 TL	0.9873	0.9874	0.9873	0.9641	0.9647	0.9641	0.9817	0.9819	0.9817
IoT-RiceMobileNet	0.9891	0.9891	0.9891	0.9878	0.9879	0.9878	0.9925	0.9926	0.9925

Note: TL = Transfer Learning. For models trained with checkpoint selection, training and validation metrics were reported from the epoch corresponding to the lowest validation loss, while test metrics were computed using the selected best-performing checkpoint.

IoT-RiceMobileNet achieved the best overall performance on the multi-source dataset, with 98.91% training accuracy, 98.78% validation accuracy, and 99.25% test accuracy. It also achieved the highest test precision and recall, with values of 99.26% and 99.25%, respectively. ResNet50 TL ranked second in test performance, achieving 98.17% test accuracy, 98.19% test precision, and 98.17% test recall. These results demonstrate that IoT-RiceMobileNet maintained strong generalization on the heterogeneous multi-source dataset while preserving a lightweight architecture suitable for real-time IoT deployment. Among the remaining models, the scratch-trained VGG16 achieved 94.04% test accuracy, outperforming ResNet50, VGG16 TL, InceptionV3 TL, InceptionV3, MobileNetV2, MobileNetV2 TL, and the baseline CNN. ResNet50 achieved 93.29% test accuracy, while VGG16 TL, InceptionV3 TL, InceptionV3, MobileNetV2, and MobileNetV2 TL achieved 92.14%, 88.14%, 83.81%, 74.25%, and 72.90% test accuracy, respectively. The baseline CNN achieved 64.84% test accuracy, highlighting the limitations of shallow architectures for complex multi-class rice disease classification.

**Fig 10:**
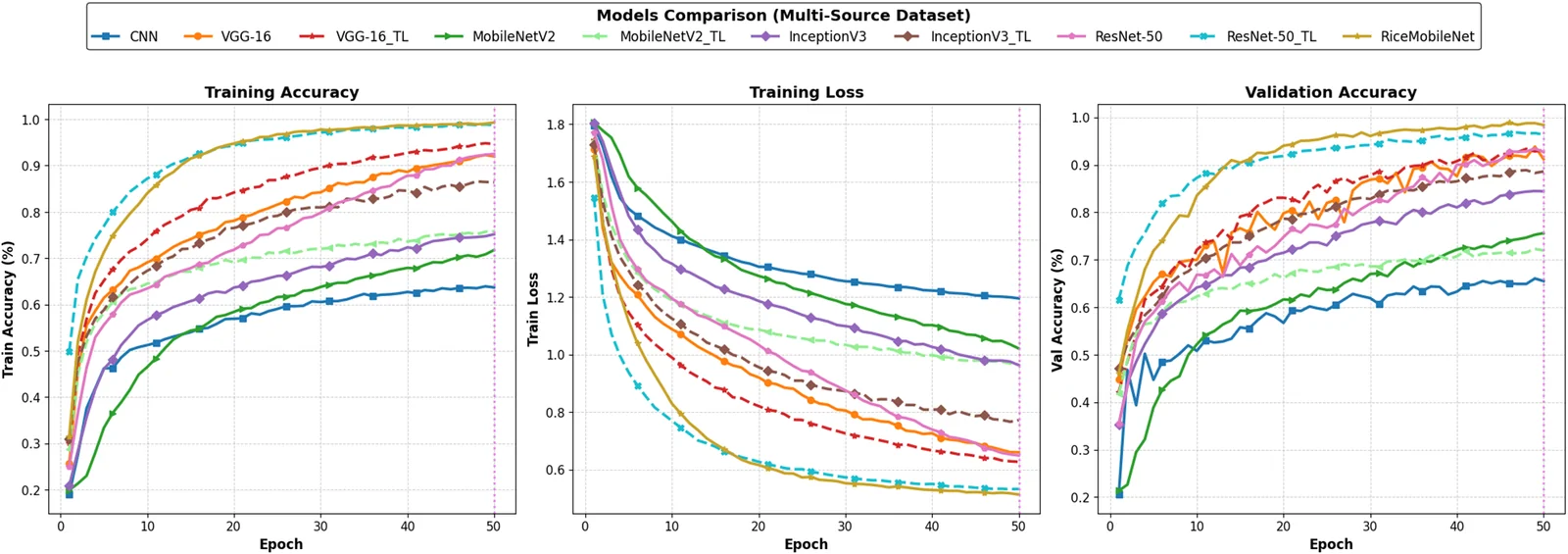
Model-wise training and validation performance across all architectures on the multi-source dataset. The figure compares training accuracy, training loss, and validation accuracy over 50 epochs. Models were trained using early stopping and checkpoint selection based on validation loss.

Overall, the results demonstrate that IoT-RiceMobileNet maintains consistently high classification performance across both the constructed RLD dataset and the more challenging multi-source dataset while preserving a lightweight architecture suitable for real-time IoT deployment, as discussed in the next subsection 17. Fig 10 shows the convergence behavior and learning stability during training.

### Model complexity and storage efficiency

Table 6 compares the computational complexity, storage requirement, and inference efficiency of the evaluated models. IoT-RiceMobileNet contains 2.418M total parameters, of which 1.876M are trainable, and requires only 9.501 MB of storage for the full model. It operates at 0.670 G FLOPs with an inference latency of 11.741 ms, corresponding to 85.17 FPS. Since a higher FPS indicates faster inference throughput, these results demonstrate that the proposed model is compact and suitable for real-time IoT-based deployment.

**Table 6:** Comparison of computational complexity and inference efficiency of different models.

Model	Parameters (M)	Trainable Params (M)	Model Size (MB)	Weight Size (MB)	FLOPs (G)	Inference Time (ms)	FPS
CNN	2.826	2.826	10.787	10.784	0.728	8.517	117.400
VGG16	14.980	14.980	57.165	57.156	30.694	80.342	12.450
VGG16 TL	14.980	7.345	57.165	57.156	30.694	64.248	15.560
MobileNetV2	2.232	2.232	8.779	8.746	0.652	16.293	61.380
MobileNetV2 TL	2.232	0.420	8.779	8.746	0.652	8.498	117.700
InceptionV3	21.798	21.798	83.508	83.470	5.709	46.761	21.390
InceptionV3 TL	21.798	6.089	83.508	83.470	5.709	31.162	32.090
ResNet50	23.520	23.520	90.050	90.025	8.263	50.110	19.960
ResNet50 TL	23.520	14.977	90.050	90.025	8.263	43.983	22.740
IoT-RiceMobileNet	2.418	1.876	9.501	9.466	0.670	11.741	85.170

Note: TL = Transfer Learning; FPS = frames per second; FLOPs = floating-point operations.

Compared with the baseline MobileNetV2, IoT-RiceMobileNet introduces only a marginal increase in complexity, from 2.232M to 2.418M parameters, 8.779 MB to 9.501 MB full-model size, and 0.652 to 0.670 GFLOPs. This small increase is justified by the improved classification performance reported in the previous section. Compared with heavier architectures, the proposed model uses approximately 6.2×, 9.0×, and 9.7× fewer parameters than VGG16, InceptionV3, and ResNet50, respectively. Its full-model size is also substantially smaller than VGG16, InceptionV3, and ResNet50, which require 57.165 MB, 83.508 MB, and 90.050 MB, respectively. In terms of inference efficiency, IoT-RiceMobileNet achieves 85.17 FPS, compared with 12.45 FPS for VGG16, 22.74 FPS for ResNet50 TL, and 32.09 FPS for InceptionV3 TL. Its inference latency is approximately 6.8× lower than VGG16 and 3.7× lower than ResNet50 TL. Although MobileNetV2 TL achieves higher throughput, IoT-RiceMobileNet provides a better balance between classification performance and computational efficiency. The slightly higher latency compared with MobileNetV2 TL is mainly due to the additional convolutional refinement layers introduced in the proposed architecture.

Overall, IoT-RiceMobileNet achieves a favorable trade-off among classification performance, model compactness, and inference efficiency, supporting its practical deployment on resource-constrained IoT platforms for rice leaf disease detection.

### Cross-validation strategy

To assess the stability and split-level generalization capability of IoT-RiceMobileNet, stratified 5-fold and 10-fold cross-validation were performed on the constructed RLD dataset. Stratification was used to preserve the original class distribution in each fold. Accuracy, precision, recall, and F1-score were recorded for each fold, and the mean ($\bar{x}$) and standard deviation (*s*) were computed across folds to quantify average performance and variability. The 95% confidence interval (CI) was estimated using the t-distribution as follows:


$$\begin{array}{c} CI_{95\%}=\bar{x}\pm t_{0.975,\;n-1}\left(\frac{s}{\sqrt{n}}\right) \end{array}$$ (8)

where $\bar{x}$ denotes the mean fold score, *s* denotes the standard deviation, *n* is the number of folds, and $t_{0.975,\;n-1}$ is the critical t-value at the 95% confidence level.

Table 7 summarizes the cross-validation performance of IoT-RiceMobileNet on the constructed RLD dataset. In 5-fold cross-validation, the model achieved a mean accuracy of 98.04±0.57% and a mean F1-score of 98.04±0.58%, with 95% confidence intervals of [97.33%, 98.75%] and [97.32%, 98.75%], respectively. In 10-fold cross-validation, the model achieved a mean accuracy of 98.13±0.85% and a mean F1-score of 98.13±0.84%, with 95% confidence intervals of [97.52%, 98.73%] and [97.52%, 98.73%], respectively. The low standard deviations and narrow confidence intervals indicate stable performance across different stratified data partitions. For each cross-validation fold, data splitting was performed before augmentation, and augmentation was applied only to the training subset of that fold to prevent data leakage between training and validation samples. Additionally, cross-validation for multi-source datasets can be found in Appendix A.

**Table 7:** Performance of IoT-RiceMobileNet on the constructed RLD dataset using 5-fold and 10-fold cross-validation.

5-Fold Cross-Validation				
Fold	Accuracy	Precision	Recall	F1-Score
1	0.980099502	0.980065303	0.980099502	0.980069341
2	0.983416252	0.983421359	0.983416252	0.983386315
3	0.988391376	0.988664137	0.988391376	0.988411042
4	0.975933610	0.976353630	0.975933610	0.975831359
5	0.974273859	0.974393447	0.974273859	0.974191562
$\bar{x}$ ± *s*	98.04 ± 0.57%	98.06 ± 0.57%	98.04 ± 0.57%	98.04 ± 0.58%
95% CI	[97.33%, 98.75%]	[97.35%, 98.77%]	[97.33%, 98.75%]	[97.32%, 98.75%]
**10-Fold Cross-Validation**				
**Fold**	**Accuracy**	**Precision**	**Recall**	**F1-Score**
1	0.971807629	0.972409963	0.971807629	0.971665109
2	0.986733002	0.986875182	0.986733002	0.986730805
3	0.981757877	0.981917996	0.981757877	0.981742263
4	0.980099502	0.980251945	0.980099502	0.980124999
5	0.986733002	0.986999298	0.986733002	0.986721840
6	0.988391376	0.988843318	0.988391376	0.988378290
7	0.985074627	0.985400422	0.985074627	0.985085518
8	0.961857380	0.963429188	0.961857380	0.962075218
9	0.988372093	0.988510490	0.988372093	0.988395469
10	0.981727575	0.981878587	0.981727575	0.981772248
$\bar{x}$ ± *s*	98.13 ± 0.85%	98.17 ± 0.81%	98.13 ± 0.85%	98.13 ± 0.84%
95% CI	[97.52%, 98.73%]	[97.59%, 98.74%]	[97.52%, 98.73%]	[97.52%, 98.73%]

Note: $\bar{x}$ = mean; *s* = standard deviation; CI = confidence interval.

### IoT system deployment and field validation

**Fig 11:**
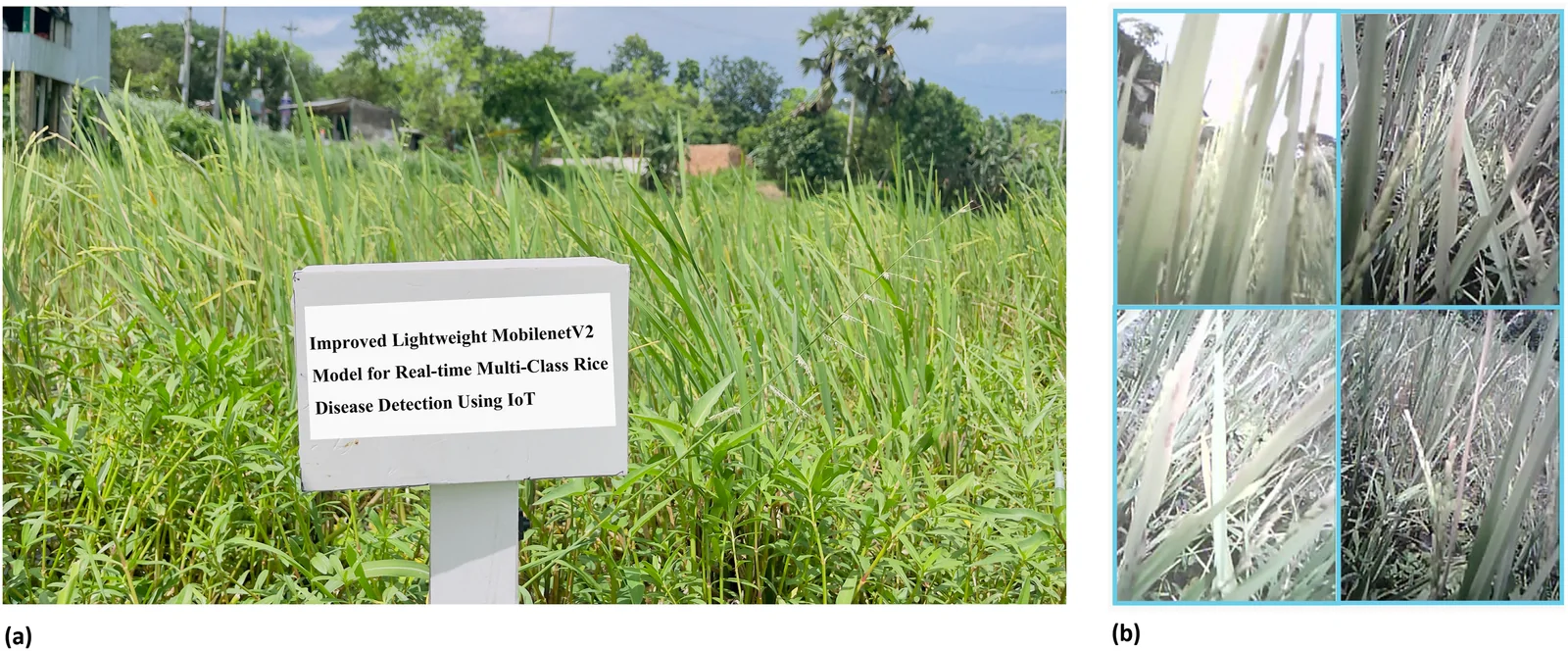
Real-time field testing of the IoT-RiceMobileNet system in an agricultural environment. (a): Physical hardware setup. (b): Real-time field image captured during testing.

**Fig 12:**
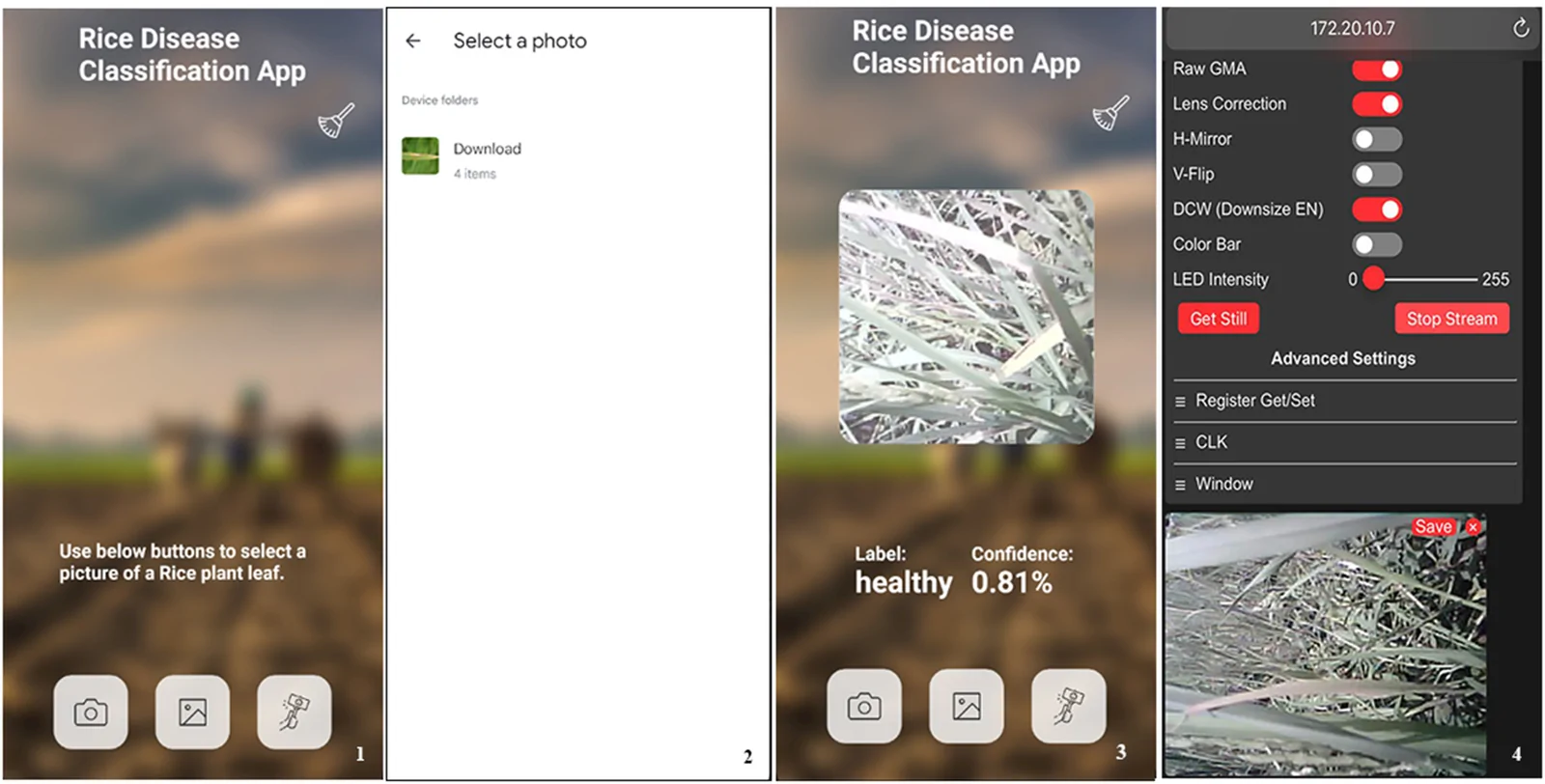
Android application interface for the IoT-RiceMobileNet system. The interface enables real-time rice disease prediction through image upload and model inference.

The proposed IoT-RiceMobileNet system was deployed using a cloud-based inference architecture that connects an ESP32-CAM device, a React Native Android application, and a FastAPI server hosted on an AWS EC2 instance running Ubuntu 22.04. The ESP32-CAM and mobile application capture rice leaf images and transmit them as JPEG files through multipart/form-data HTTP POST requests to the /predict API endpoint. Field connectivity was provided through a smartphone hotspot, requiring no dedicated networking infrastructure. The physical hardware setup, real-time field image capture, and Android application interface are shown in Figs 11 and 12.

Upon receiving an image, the server validates the input and applies the same preprocessing pipeline used during training, including resizing to 224×224 pixels, RGB conversion, and normalization. The preprocessed image is then passed to the deployed PyTorch-based IoT-RiceMobileNet model, where inference is performed in evaluation mode using torch.no_ grad(). The output logits are converted into class probabilities using softmax, and the predicted disease class with its confidence score is returned to the client as a JSON response. The main communication and deployment settings are summarized in Table 8.

**Table 8:** Communication and deployment summary of the IoT-based rice disease detection system.

Parameter	Details
Protocol	HTTP REST API via FastAPI
API endpoints	/predict (POST), /ping (GET)
Payload format	multipart/form-data with JPEG image upload
Response format	JSON with predicted class label and confidence score
Server framework	FastAPI with PyTorch inference backend
Cloud deployment	AWS EC2 running Ubuntu 22.04
IoT connectivity	Wi-Fi through smartphone hotspot
Mobile client	React Native Android application
Preprocessing	Resize to 224×224, RGB conversion, normalization
Mean end-to-end latency	516 ms (SD: 23.1 ms, *n* = 10)

End-to-end latency was measured over ten independent trials using client-side timestamps recorded before image transmission and after receiving the server response. The measured latency includes image upload, preprocessing, model inference, and response delivery:


$$\begin{array}{c} \text{Latency (ms)}=T_{\text{received}}-T_{\text{sent}}. \end{array}$$ (9)

The system achieved an average response time of 516 ms with a standard deviation of 23.1 ms, indicating consistent sub-second performance. Although IoT-RiceMobileNet can be exported to ONNX and TensorFlow Lite formats, cloud-based inference was adopted to reduce mobile application size, simplify model updates, and improve compatibility across heterogeneous devices. A pilot field validation was conducted using ten rice leaf images captured under real agricultural conditions through the ESP32-CAM device and the mobile application. The system processed all submissions without communication failure and correctly classified seven of the ten field samples, indicating preliminary feasibility under real agricultural conditions. Although this pilot evaluation is limited in scale, it provides preliminary evidence of practical field usability under real deployment conditions.

The battery operating time of the ESP32-CAM device, powered by an 11.1 V, 1000 mAh LiPo battery through an LM2596S buck converter, was estimated using the measured current consumption and the converter efficiency characteristics reported in the manufacturer’s datasheet [49]. The average operating current, determined from the measured current consumption during deep sleep, Wi-Fi idle, and active transmission over a 30-second image capture interval, was approximately 100 mA. Accordingly, the expected operating time was calculated as


$$\begin{array}{c} t=\frac{9.77\;Wh}{0.5\;W}\approx 19.5\;h \end{array}$$ (10)

This corresponds to approximately two 8-hour field sessions per charge, supporting sustained field operation under typical agricultural monitoring scenarios.

### Proposed model detailed analysis and comparison

The IoT-RiceMobileNet model achieved test accuracies of 99.19% and 99.25% on the constructed RLD dataset and the multi-source dataset, respectively. The multi-source dataset was used as an additional heterogeneous benchmark, where all models were trained, validated, and tested using the same experimental protocol. Table 9 reports the training accuracy, validation accuracy, and test performance metrics of IoT-RiceMobileNet across both datasets.

**Table 9:** Overall performance of IoT-RiceMobileNet across the constructed RLD and multi-source benchmark datasets.

Dataset	Train Acc.	Val. Acc.	Test Acc.	Prec.	Rec.	F1-score
Constructed RLD dataset	98.73%	97.85%	99.19%	99.20%	99.19%	99.19%
Multi-source dataset	98.91%	98.78%	99.25%	99.26%	99.25%	99.26%

**Fig 13:**
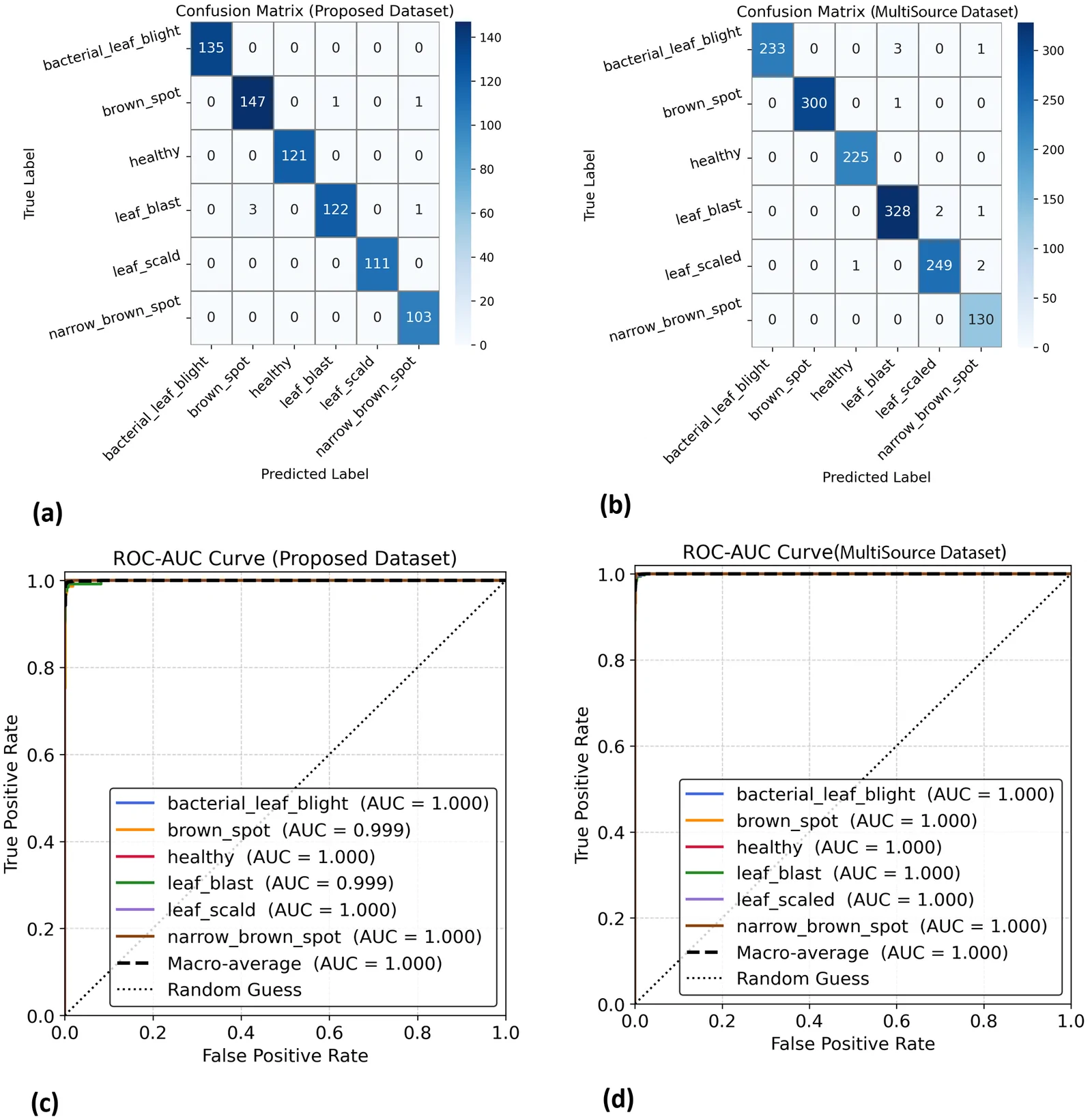
Performance comparison of IoT-RiceMobileNet using confusion matrices and ROC-AUC curves across datasets. (a): Confusion matrix for the constructed RLD dataset. (b): Confusion matrix for the multi-source dataset. (c): ROC-AUC curve for the constructed RLD dataset. (d): ROC-AUC curve for the multi-source dataset.

**Fig 14:**
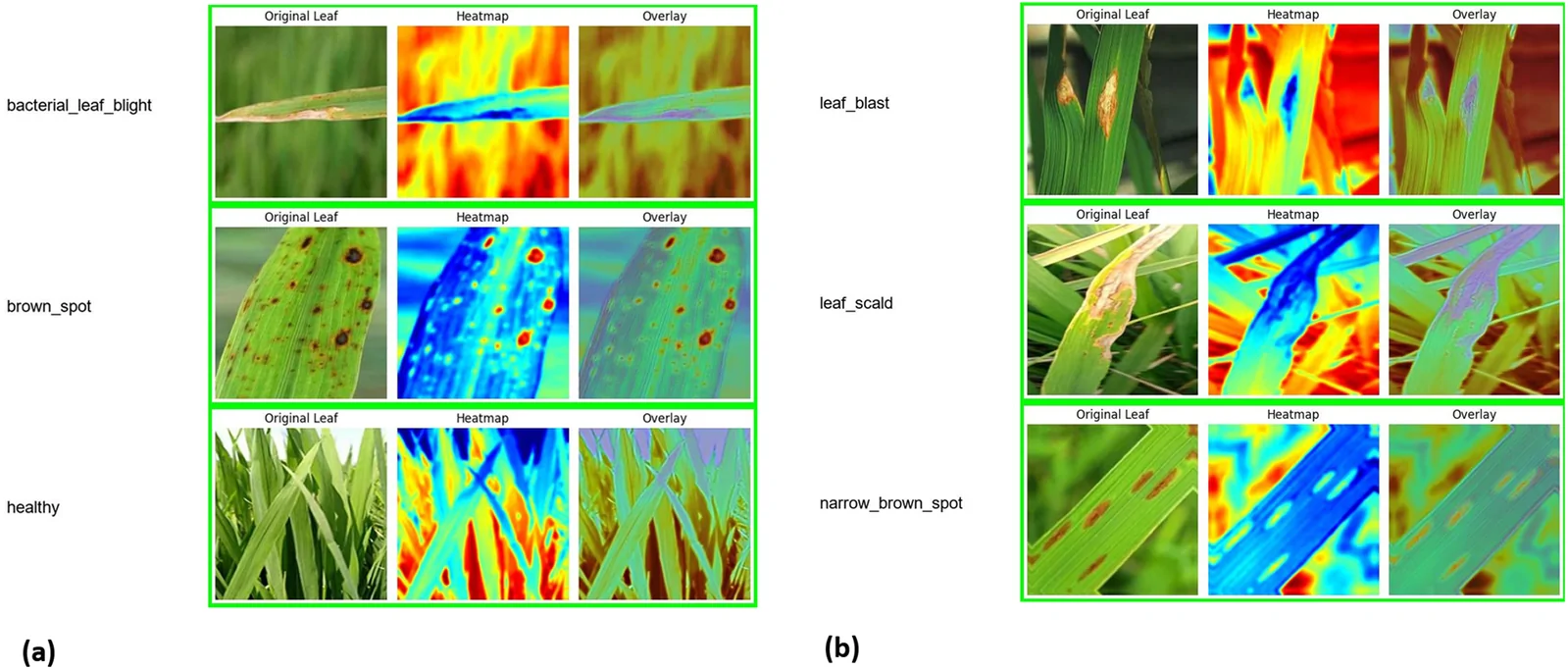
Grad-CAM visualizations showing model attention regions across rice leaf conditions. A: Diseased samples. B: Healthy samples. Warmer colors indicate higher influence on classification decisions.

The confusion matrices and ROC-AUC curves in Fig 13 further show the classification behavior of the model across all six rice leaf classes. Grad-CAM visualizations in Fig 14 show that the model mainly focuses on visible lesions and affected leaf regions for diseased samples. For healthy samples, the attention maps are more diffuse, reflecting the absence of localized disease symptoms. These visualizations support the interpretability of the proposed model.

Table 10 compares IoT-RiceMobileNet with recent rice disease detection studies. Although these studies differ in datasets, class numbers, and experimental settings, IoT-RiceMobileNet achieved a competitive accuracy of 99.19% across six classes while also supporting an end-to-end IoT deployment framework.

**Table 10:** Comparison of IoT-RiceMobileNet with existing rice leaf disease detection methods.

Authors	Method	Classes	Accuracy
[50]	Inception + ResNetV2 + CNN	4	95.67%
[51]	Faster R-CNN + EfficientNet-B0	3	96.43%
[27]	DenseNet169 + MLP	3	97.68%
[33]	InceptionV3 + CNN	3	94.48%
[31]	StyleGAN2-ADA + Faster-RCNN + SSD	4	93.00%
[40]	SVM + CNN	5	91.45%
[28]	RegNet transfer learning	12	96.80%
[52]	YOLOv8n-MBS + C2f_MSEC + BiFPN + SLDH	3	93.90%
[37]	RGC-YOLO	4	93.20%
[29]	RDRM-YOLO	4	94.30%
[30]	SANET (ResNet + CNN + KAM)	4	98.71%
**IoT-RiceMobileNet**	**MobileNetV2 + CNN**	**6**	**99.19%**

Note: Accuracy values are reported from the respective studies. Direct comparison may be affected by differences in datasets, disease classes, preprocessing strategies, and experimental settings.

## Discussion

The IoT-RiceMobileNet model demonstrates viable deployment of lightweight, real-time disease detection in agricultural environments with limited computing resources. The model achieved 99.19% test accuracy on the constructed RLD dataset and 99.25% test accuracy on the multi-source dataset, while maintaining 2.418M parameters, 9.501 MB model size, and 85.17 FPS, demonstrating strong performance across both evaluation settings. The results support the objective of this study: developing a lightweight, accurate, and deployable rice leaf disease detection framework for smart agriculture. Transfer learning effectiveness varied across architectures; however, IoT-RiceMobileNet achieved the strongest test-set performance across both evaluation settings while maintaining a substantially smaller model size than heavier transfer learning baselines. ResNet50 TL generally ranked second in the updated comparative experiments, but its larger parameter count and storage requirement make it less suitable for resource-constrained IoT deployment. The stable convergence behavior suggests that the selected optimization strategy and learning-rate scheduling contributed to effective model training.

Although IoT-RiceMobileNet achieved strong performance across the constructed RLD dataset and the multi-source benchmark dataset, several limitations remain. The field evaluation was limited to a small number of real-time trials and should be interpreted as a preliminary feasibility assessment rather than a large-scale deployment validation. Moreover, 95% confidence intervals are reported for the cross-validation experiments, formal pairwise statistical significance tests between IoT-RiceMobileNet and the baseline models were not conducted, as the baselines were evaluated using a single best-checkpoint protocol. Consequently, the observed performance differences, while consistent, are not accompanied by significance-based confirmation; pairwise testing, such as McNemar’s test on test-set predictions or paired t-tests across cross-validation folds, is left to future work. Furthermore, while the Model development section explains the rationale for each architectural component, a controlled ablation isolating their individual contributions was not conducted, leaving their specific quantitative impact uncharacterized as a limitation left to future work. In addition, the implemented prototype relied on cloud-based inference, which may be affected by network availability and latency in real-world agricultural settings, particularly in rural regions with poor or intermittent connectivity where reliance on a remote server may reduce reliability. Real-world farm environments vary significantly in lighting, image quality, and environmental conditions. Extensive field validation across regions and seasons is needed to assess practical performance. Additionally, expansion to more disease categories and integration with agricultural decision-support platforms would enhance utility.

Future work should focus on expanding the dataset across additional rice varieties, geographic regions, and environmental conditions; optimizing the model through quantization and pruning to support offline on-device edge deployment for low-connectivity regions; integrating environmental sensor data; improving explainability using SHAP or attention-based methods; conducting a controlled ablation study to quantify the contribution of each architectural component; performing pairwise statistical significance testing across models; and conducting larger field trials to further assess robustness under real deployment conditions.

## Conclusion

This study introduces IoT-RiceMobileNet, a compact deep learning system for real-time rice leaf disease detection that achieved 99.19% test accuracy on the constructed RLD dataset while maintaining computational efficiency for mobile and IoT-based deployment. Additional evaluation on a heterogeneous multi-source dataset of 14,758 images across six classes further demonstrated strong generalization, with IoT-RiceMobileNet achieving 99.25% test accuracy. Grad-CAM analysis indicates that the model focuses on disease-relevant leaf regions during classification, while the end-to-end IoT integration supports the practical feasibility of the proposed system for agricultural deployment. The proposed architecture requires substantially fewer parameters than conventional deep learning models, enabling scalable and low-cost disease monitoring in resource-constrained farming communities. This work contributes toward practical digital agriculture by supporting scalable, low-cost rice disease monitoring in resource-constrained farming communities. With further field validation and dataset expansion, IoT-RiceMobileNet can serve as a practical foundation for accessible rice disease monitoring and decision support in precision agriculture.

## Supporting information

S1 Appendix Cross-validation results for the multisource dataset. (PDF)
