# Supplementary material for: IoT-RiceMobileNet: An improved lightweight MobileNetV2 Model for real-time multi-class rice disease detection using IoT
Source: PLoS One. 2026 Sep 15;21(9):e0356383. doi: 10.1371/journal.pone.0356383 (PMC13577542; doi:10.1371/journal.pone.0356383)
Supplement: Supplementary file 1 [file pone.0356383.s001.pdf]

S1 Appendix

Cross-validation on multi-source dataset

Table 1: **Performance of IoT-RiceMobileNet on the Multisource dataset using 5-fold and 10-fold cross-validation.**

**5-Fold Cross-Validation**				
**Fold**	**Accuracy**	**Precision**	**Recall**	**F1-Score**
1	0.979678239	0.979648232	0.979678239	0.979653403
2	0.981787378	0.981826047	0.981787378	0.981791351
3	0.982634477	0.982893518	0.982634477	0.982676762
4	0.982210928	0.982956603	0.982210928	0.982364653
5	0.979246082	0.979487846	0.979246082	0.979269230
$\bar{x} \pm s$	98.11 ± 0.15%	98.14 ± 0.17%	98.11 ± 0.15%	98.12 ± 0.16%
95% CI	[97.92%, 98.30%]	[97.93%, 98.35%]	[97.92%, 98.30%]	[97.92%, 98.31%]

**10-Fold Cross-Validation**				
**Fold**	**Accuracy**	**Precision**	**Recall**	**F1-Score**
1	0.984758679	0.984974406	0.984758679	0.984797712
2	0.981371719	0.981379208	0.981371719	0.981367183
3	0.987298899	0.987491340	0.987298899	0.987325846
4	0.983911939	0.984025461	0.983911939	0.983902624
5	0.985605419	0.986321526	0.985605419	0.985727626
6	0.990685859	0.990817273	0.990685859	0.990692904
7	0.988135593	0.988149169	0.988135593	0.988139538
8	0.987288136	0.987331194	0.987288136	0.987286890
9	0.982203390	0.982609530	0.982203390	0.982261116
10	0.986440678	0.986618025	0.986440678	0.986458369
$\bar{x} \pm s$	98.58 ± 0.28%	98.60 ± 0.28%	98.58 ± 0.28%	98.58 ± 0.28%
95% CI	[98.38%, 98.78%]	[98.40%, 98.80%]	[98.38%, 98.78%]	[98.38%, 98.78%]

Note: $\bar{x}$ = mean; s = standard deviation; CI = confidence interval.

Table 1 summarizes the cross-validation performance of IoT-RiceMobileNet on the multisource dataset. In 5-fold cross-validation, the model achieved a mean accuracy of $98.11 \pm 0.15\%$ and a mean F1-score of $98.12 \pm 0.16\%$, with 95% confidence intervals of [97.92%, 98.30%] and [97.92%, 98.31%], respectively. In 10-fold cross-validation, the model achieved a mean accuracy of $98.58 \pm 0.28\%$ and a mean F1-score of $98.58 \pm 0.28\%$, both with a 95% confidence interval of [98.38%, 98.78%]. The low standard deviations and narrow confidence intervals indicate stable performance across different stratified data partitions. For each cross-validation fold, data splitting was performed before augmentation, and augmentation was applied only to the training subset of that fold to prevent data leakage between training and validation samples.
